# Future vision for the quality assurance of oncology clinical trials

**DOI:** 10.3389/fonc.2013.00031

**Published:** 2013-03-15

**Authors:** Thomas J. FitzGerald, Maryann Bishop-Jodoin, Walter R. Bosch, Walter J. Curran, David S. Followill, James M. Galvin, Richard Hanusik, Steven R. King, Michael V. Knopp, Fran Laurie, Elizabeth O'Meara, Jeff M. Michalski, Joel H. Saltz, Mitchell D. Schnall, Lawrence Schwartz, Kenneth Ulin, Ying Xiao, Marcia Urie

**Affiliations:** ^1^Quality Assurance Review CenterLincoln, RI, USA; ^2^Department of Radiation Oncology, University of Massachusetts Medical SchoolWorcester, MA, USA; ^3^Department of Radiation Oncology, Washington University School of MedicineSt. Louis, MO, USA; ^4^Department of Radiation Oncology, Emory University School of MedicineAtlanta, GA, USA; ^5^Radiological Physics Center, Department of Radiation Physics, University of Texas MD Anderson Cancer CenterHouston, TX, USA; ^6^Department of Radiation Oncology, Jefferson Medical College, Thomas Jefferson UniversityPhiladelphia, PA, USA; ^7^Radiation Therapy Oncology GroupPhiladelphia, PA, USA; ^8^American College of RadiologyPhiladelphia, PA, USA; ^9^Department of Radiology, Ohio State College of MedicineColumbus, OH, USA; ^10^Department of Biomedical Informatics, Emory UniversityAtlanta, GA, USA; ^11^Department of Radiology, Hospital of the University of PennsylvaniaPhiladelphia, PA, USA; ^12^Department of Radiology, Columbia UniversityNew York, NY, USA

**Keywords:** radiation oncology, quality assurance, oncology clinical trials, National Cancer Institute, Clinical Trials Cooperative Group Program

## Abstract

The National Cancer Institute clinical cooperative groups have been instrumental over the past 50 years in developing clinical trials and evidence-based process improvements for clinical oncology patient care. The cooperative groups are undergoing a transformation process as we further integrate molecular biology into personalized patient care and move to incorporate international partners in clinical trials. To support this vision, data acquisition and data management informatics tools must become both nimble and robust to support transformational research at an enterprise level. Information, including imaging, pathology, molecular biology, radiation oncology, surgery, systemic therapy, and patient outcome data needs to be integrated into the clinical trial charter using adaptive clinical trial mechanisms for design of the trial. This information needs to be made available to investigators using digital processes for real-time data analysis. Future clinical trials will need to be designed and completed in a timely manner facilitated by nimble informatics processes for data management. This paper discusses both past experience and future vision for clinical trials as we move to develop data management and quality assurance processes to meet the needs of the modern trial.

## INTRODUCTION

In the past 50 years, the National Cancer Institute (NCI) Clinical Trials Cooperative Group Program has generated an extraordinary legacy of ground breaking clinical and translational science; significantly influencing the standard care for oncology patients. As cancer therapy became more universally available in the mid-twentieth century, oncology specialists began discussing standards of care and how these standards could be applied in a multi-institution cooperative group format. Pre-conceived beliefs in treatment standards coupled with institution bias often limited successful uniform clinical trial execution. Over time, the concept of multi-institution clinical trial strategies matured into successful federally funded programs. The development and maturation of systemic therapy extensively influenced the importance and status of cooperative group clinical trials. Only multi-institution trials, with large study populations, could validate the role of new systemic chemotherapy strategies including dose and dose scheduling in a timely manner. Participation in clinical trials enhanced the public perception of both large and small institutions as participation suggested that the oncology skill set of the treatment program functioned at a high level. Clinical faculty functioned as clinical trial investigators and trial participation became important and influential in institution promotion processes. Financial incentives for trial participation provided the management infrastructure for the institutional trial offices. Although reimbursement did not cover full trial costs, finances from clinical trials were used to promote and facilitate participation in the clinical trials process. The cooperative group administrative structures matured as trials became more complex. Integration of privately and federally funded trial processes for the adult and pediatric groups have facilitated therapy development as clinical trials have served to validate treatment strategies. Enrolling over 25,000 patients yearly through the cooperative groups, the NCI Clinical Trials Cooperative Group Program was considered a clear and unambiguous success.

Self-renewal and re-evaluation have affected the clinical trials process. While recognizing the success of the clinical trials program, the Institute of Medicine (IOM) report of 2010 identified the need for change (Institute of Medicine of the National Academies, 2010). The report indicated that trials processes were too extended with imbedded system redundancies requiring streamlining of the trial development process and strategy. The result will be a 50% decrease in the number of cooperative groups with new emphasis on international participation and trial development through cancer center mechanisms. This will provide an economy of scale for phase 3 trials and enhanced efficiencies for completing phase 1 and 2 trials including adaptive mechanisms for study execution. An effort will be made to limit the global cost of trial development while increasing institution reimbursement for execution. These are clear and bold objectives designed to enhance thoroughness and efficiency. More than 50% of plenary discussions at our most important national and international meetings are generated from the outstanding clinical trials work. Nevertheless, change is upon us and our success will be measured and determined by how we adapt to the altered environment.

Accordingly, quality assurance (QA) in the cooperative groups will be revised. QA processes have evolved becoming a robust enterprise fluent in protocol development, credentialing, data acquisition, management, transfer, and international real-time digital imaging and radiation therapy (RT) object data review and feedback. Each of the QA groups, including the imaging and RT components of study groups, has approached credentialing and QA activities in various ways and each has achieved the similar outcomes. Developing these programs have been important and will continue to be important in this new network which will be renamed the Imaging/Radiation Oncology Core Group (IROC). This group will be administered by the American College of Radiology (ACR) and integrate the imaging strengths of the ACR core lab of the ACR Imaging Network (ACRIN), the imaging core lab of the Cancer and Leukemia Group B (CALGB) at Ohio State/Wright Imaging Center, the Quality Assurance Review Center (QARC), and the radiation oncology strengths of the Radiological Physics Center (RPC), the Image-Guided Therapy Center (ITC; Washington University, St. Louis), the Radiation Therapy Oncology Group Core Lab (RTOG QA Center), and QARC.

## PROTOCOL DEVELOPMENT

The QA centers have developed standardized RT protocol templates based upon the radiation technology needs and protocol objectives. Early involvement in the protocol development process enables timely discussion of the required credentialing, data items to be collected and imaging/radiotherapy review(s) to be performed. Continued collaboration with the imaging and RT committees in each cooperative group is critical to producing a well-written protocol at the initiation of a clinical trial that will efficiently and cost-effectively answer the study questions. Members of the QA centers participate in all discipline and disease-based committees of the cooperative groups in order to facilitate the incorporation of QA processes into the clinical trial charter. Members of the QA centers maintain strong relationships with both administrative and data management cores of each cooperative group in order that QA processes are imbedded in the clinical trial charter. The NCI review of the protocol is thorough and serves to further promote consistent standards for QA.

## CREDENTIALING

### RT CREDENTIALING

Credentialing institutions for participation in clinical trials has been a cornerstone feature of the QA process for decades and will remain very important moving forward. RT credentialing began as a process to validate the physical dose delivered on each therapy unit and provided a planning test case (benchmark) generic to each protocol. The intent was and remains to ensure that planning and treatment delivery can be uniform and consistent with standards. As treatment technologies and therapy execution became more complex, credentialing mechanisms have likewise adapted to meet this need including strategies for disease-based intensity modulation, motion management, and radiosurgery to multiple targets. Contrary to the opinion of those less involved with the QA process, the IROC centers in both imaging and RT have integrated their efforts well over the past several years providing seamless credentialing in physical dose, image fusion, target volume definition, and advanced technology treatment execution. Moving forward, these processes will need to become fully transparent to the clinical trials enterprise with each new cooperative group participating in the credentialing process in a symbiotic and synergistic manner. Each cooperative group will have slightly different needs for credentialing and thus a tiered system will likely be used specific for each protocol moving forward. In studies where RT is part of the study but not a specific study endpoint, credentialing may be at a level commensurate with the technology needed for the study. For example, a lung cancer study in SWOG may require intensity-modulated RT (IMRT) credentialing validation coupled with a motion management questionnaire along with image credentialing for positron emission tomography/computerized tomography (PET/CT) fusion into radiation planning computer tomography. This will be a different mechanism than a multi-group trial evaluating the use of stereotactic RT lung and liver metastasis that will require more advanced and highly treatment specific credentialing for motion management. IROC will need to work with each cooperative group to facilitate a credentialing strategy that meets the specific needs of each protocol without limiting study accrual objectives. Pediatrics will remain unique and likely require more real-time review of objects and treatment plans as even experienced treatment teams appreciate validation of plan intent for study compliance. Pediatric patients are treated in a geographically diverse manner with each cancer center treating a limited number of patients in each disease site every year; therefore, with the exception of phase 1 and technology-specific protocols, it will remain less likely that a limited institution study can be performed in the pediatric community, especially in the field of rare tumors.

### IMAGING CREDENTIALING

American College of Radiology Imaging Network has provided a strong infrastructure in developing a credentialing strategy for radiology and the cancer centers using multiple technologies including both metabolic and anatomic imaging tools. The CALGB imaging core service has a credentialing strategy in alignment with ACRIN and has provided very good data on PET QA in clinical trials. Pediatrics will bring a separate problem set for credentialing including accelerated imaging needs in ultrasound and magnetic resonance (MR) that will be managed with the imaging committee of the Children's Oncology Group (COG). Metabolic imaging including spectroscopy and dynamic contrast technology are under evaluation in clinical trials and may become important biomarkers in selected disease-based programs. ACRIN and the CALGB imaging core center are poised and well equipped to provide a clear imaging credentialing infrastructure. Together with QARC and the resources of the ACR, the group has considerable strength in providing the education and infrastructure to validate imaging interpretation and the tools to ensure study interpretations are uniform and compliant to study objectives. Similar to RT, there is a need to establish a tiered credentialing mechanism that may be study specific for institutions participating in clinical trials with advanced technology imaging techniques. Potential strategies include periodic credentialing and validation of image acquisition processes and quality during a clinical trial.

As clinical trials move toward more international participation, credentialing will play a very important role. In addition to the initial credentialing, periodic re-credentialing is important aspect of the continued QA. Currently, in RT, re-credentialing is required when an institution/RT department changes their planning system or when a new benchmark is established for advanced technology therapy. As we move forward and develop more precise benchmarks to validate the contour of structures for both target volume definition and normal tissue structures, there may be more than one group of investigators within each institution to manage both disease-based treatment and pediatrics. Therefore, renewing the credentials for investigators and institutions on a periodic basis will insure that quality processes are maintained once credentials have been acquired.

## INFORMATICS – DATA ACQUISITION/MANAGEMENT

The informatics platforms throughout the IROC are comprehensive infrastructures providing the foundation for the clinical trial support services. Currently, study data resides in the cooperative group data centers, tissue banks, and QA centers and is integrated as needed through each protocol and cooperative group relationship. The QARC program utilizes a relational database, and is the center of QARC's infrastructure. Validation was completed in 2008 making the database fully 21 Code of Federal Regulations (CFR) Part 11 compliant. Processes and controls are in place to maintain validation as development for new programs and technologies are a constant. Streamlined processes enable the support of multiple data acquisition methods. Efforts are underway within IROC to achieve interoperability and integration of the existing informatics systems.

## CASE REVIEW

**FIGURE 1:**
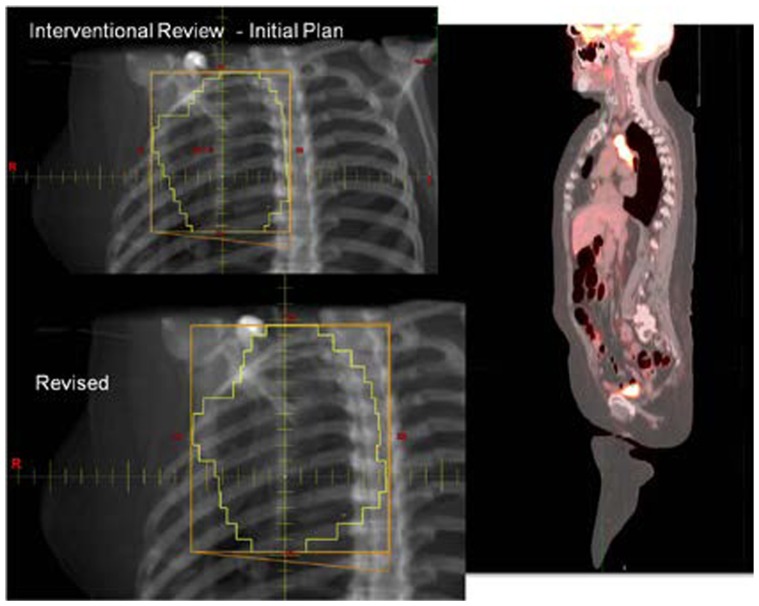
**Case submitted for interventional review demonstrating necessary volume revision**.

**FIGURE 2:**
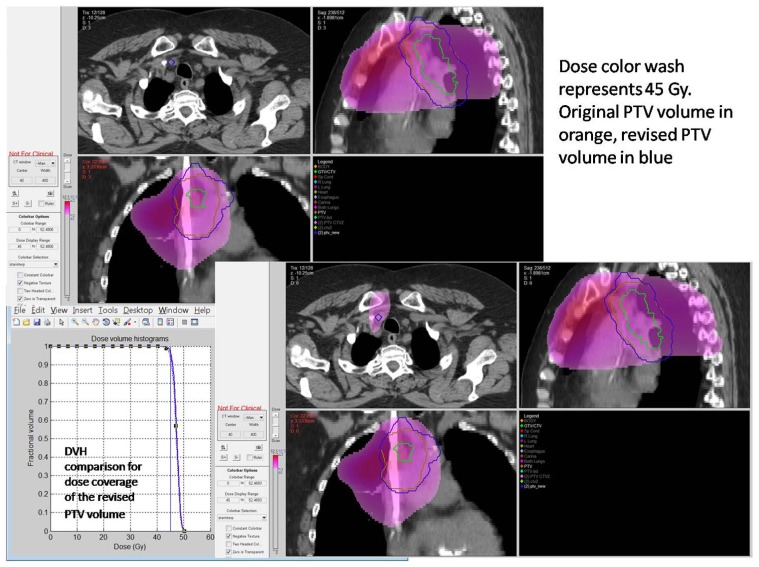
**Case submitted for interventional review in Figure 1 demonstrating improved dose coverage**.

With the increasing use of advanced technology RT, there has been an accelerated use of real-time review of both imaging and RT treatment objects in the design and execution of clinical trials. The objective is to insure compliance to study target volumes and intended therapy dose as well as provide central review of imaging objects for study execution. All current QA centers offer a mechanism for real-time (rapid) review of therapy objects and offer this as a mechanism for intervention. The clinical trial benefits of real-time review have been well demonstrated by several pediatric and adult studies (Tebbi et al., 2006; Wolden et al., 2009; Peters et al., 2010). Real-time review accomplishes several objectives: it establishes both a platform to review submitted information for quality and facilitates compliance to study objectives including clinical stage. This ensures that the patient is eligible for the specified study and that the data are complete and can be used for trial analysis. The review can identify incomplete datasets and provide corrective action prior to patient treatment (**Figures 1** and **2**).

Real-time and interventional review is criticized at multiple levels. Those outside of the clinical trials system suggest that processes developed for real-time review are far too elaborate and do not reflect actual clinical practice. There is an assumption that deviations on study will be evenly distributed across study arms and, accordingly, deviations will not influence study outcome. We have seen many examples in both pediatric and adult studies that clinical trial deviations have direct consequence on trial outcome and deviations in image interpretation and RT can have profound impact on studies where neither imaging or RT were the study endpoints. The influence of real-time review may not be obvious to a casual observer; however, the impact on clinical trial execution can be profound. A retrospective review of image objects at the time of relapse in a high-risk pediatric medulloblastoma trial revealed a number of patients with reported relapse/progression actually had changes most compatible with treatment effect, not relapse. Further more than 10% of the patients enrolled are considered ineligible due to artifacts (motion) or limitations in spinal imaging. Accrual objectives are negatively influenced by ineligible patients and there is a cost associated with ineligible patients. Real-time intervention can limit patients lost to study process and serve to improve trial objectives.

**FIGURE 3:**
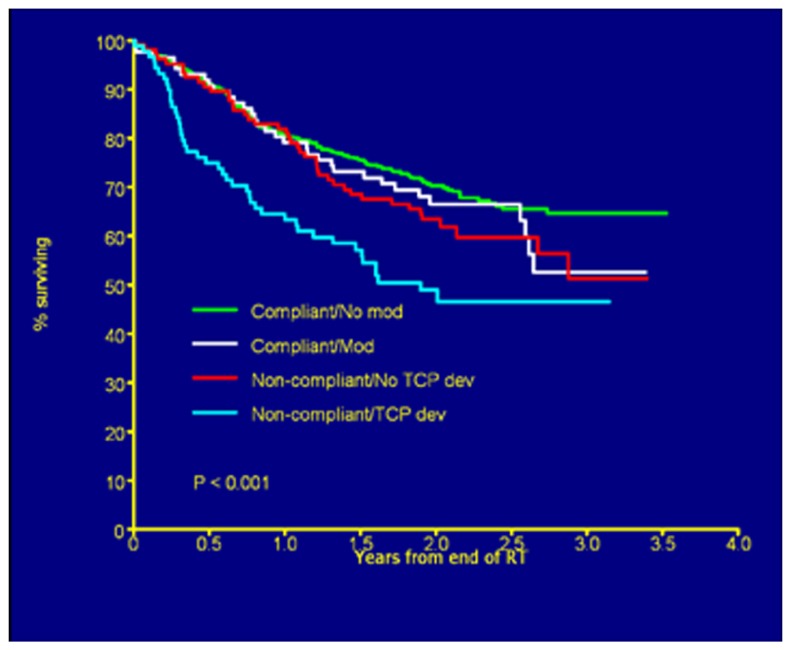
**Overall survival by deviation status.** Figure as originally published in Peters et al. (2010).

Resources for real-time review of clinical trial objects will need to be thoughtfully applied. Drawing upon lessons learned there is a relationship to the introduction of new technologies or applied technologies with study deviation. In the Wilms' tumor experience, it was not anticipated that image fusion or applied three-dimensional RT treatment objects would be difficult for the clinical trials community to assimilate into the clinical trials process. However, radiation oncologists have not been able to make the needed adjustments using modern planning tools in a treatment area that historically has been approached with established two-dimensional treatment strategies. In the HeadSTART trial, we saw deviation patterns consistent with uncertainties in the application of imaging objects into RT treatment planning systems (**Figure 3**. As time evolves, these issues are addressed through both the clinical trials process and clinical experience. History has shown that as investigators become familiar with disease-based protocol applications and consistent in executing clinical trial objectives, new technologies, and advanced imaging strategies are introduced into clinical trials that will require intervention to make certain they are applied in a protocol compliant manner. Intensity modulation and motion management are two very good examples where varied technologies need to be applied in a uniform manner for protocol compliance. The process of intervention permits uniform application of these technologies in a protocol compliant manner. Although we casually perceive these tools as the standard of care, from a clinical trials perspective we are not at a point of development where we can simply assume without intervention that these tools are used in a uniform clinical trial manner. In fact, evidence suggests otherwise. Our technologies allow us to re-invent our treatment strategies on a constant basis. The process of re-invention is diverse, complicated by varied therapy technologies and techniques supported through multiple vendors. QA processes and a real-time intervention strategy permit the appropriate degree of QA, and facilitate the assimilation of diverse platforms into a common treatment execution strategy. As platforms and disease treatment strategies mature and treatment paradigms become more consistent, adjustments can be made in the QA process to place emphasis and resources in new areas of development and examine potential controversy. In collaboration with the imaging and RT committees of each new cooperative group, the newly formed QA center will need to anticipate the likelihood for real-time intervention and write these requirements into the clinical trial charter as part of clinical trial development. It remains easier and more cost-effective to anticipate this need rather than react to an unanticipated development after a trial has been activated. Each clinical trial with RT will likely have an assigned category for real-time review, dose validation, contouring, and motion management. There will likely be trials that focus on credentialing with data collection and no real-time intervention. Others, including trials with developing technologies or difficult contouring challenges, may place heavy emphasis on real-time review of objects. These and other processes will be debated through committee and included as part of the clinical trial charter. Having established these points, there will need to be processes in place during the clinical trial operation to insure that the chosen strategy for QA is fulfilling the objectives of the study. This will be an on-going process of self-renewal and lessons learned from adaptive mechanisms that can be built into the next iteration of the clinical trial.

A secondary benefit to real-time interventional review is the fact all of the data can be reviewed at the time of patient treatment and incomplete records can be completed as part of that process.

## HISTORY AND LESSONS LEARNED

### EARLY RT QA

Quality assurance has always played a unique role in the clinical trials enterprise. The early contributions of radiation oncology medical physicists working closely with their clinician colleagues led to the development of radiation treatment QA programs that are unparalleled by other medical specialties involved in clinical trials. These early protocols that used radiation for therapy led to rigorous QA and educational programs. Compliance systems were established to verify the radiation doses received by protocol subjects and assure the participating institutions and investigators understood the technical demands for protocols that incorporated new radiation dose delivery technologies (D'Angio, 1980; Galvin et al., 1980; Kim et al., 1980). More recently, credentialing methods have been introduced to verify performance of the protocol components. A system has evolved to ensure common ground in protocol execution and to create uniform study populations for study outcome validation.

Quality assurance implies the appropriate diagnosis and staging are obtained and meet protocol standards, patient studies are acquired and interpreted in a uniform format, and the treatment is conducted in a protocol compliant manner. QA processes have changed over the 50 year cooperative group history. For the cooperative groups that originated in 1960/1970, retrospective reviews were done with hard copy data and images. There was no defined role for review intervention prior to therapy execution. RT study deviations were largely computational as there was a non-uniform approach to treatment calculation and no images were available to validate the target volume. Initially it was difficult to obtain the data and images. Over time clinical trials required data review as part of the study conduct. The timeliness and quality of data submitted for review were incorporated into institutional performance review.

In the early days of clinical trial development, retrospective data review was important but had clear limitations. Communication capabilities were not as sophisticated as they are currently, and the data influenced in the processes written for the next generation of trials made no immediate contribution to the active study. The knowledge gained from the clinical trial data reviews including lessons learned from study deviations was only recognized through generations of subsequent trials. The processes would, by default, unintentionally promote significant misinterpretation of study outcome (Weiner et al., 1997; Kachnic et al., 2011; Abrams et al., 2012).

### LESSONS LEARNED

**FIGURE 4:**
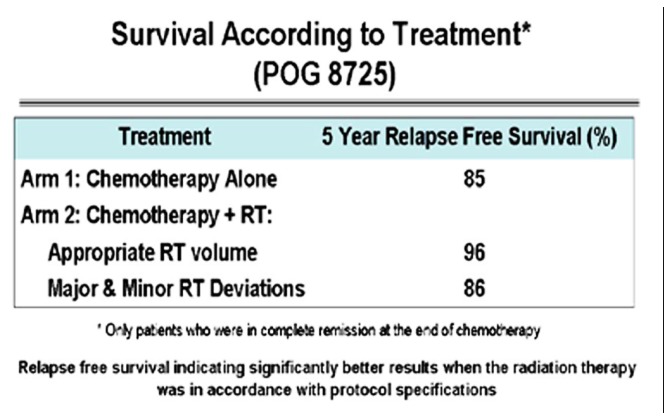
**Pediatric Oncology Group (POG) Protocol 8725 Survival according to treatment.** Figure originally published in FitzGerald et al. (2008).

A retrospective review of the Pediatric Oncology Group (POG) Protocol 8725 data objects, which included review of images demonstrating the sites of disease at presentation and review of RT treatment objects, revealed a statistically significant survival advantage to patients treated with RT in a protocol compliant manner (FitzGerald et al., 2008). This review confirmed that RT treatment delivered in a protocol compliant manner affected patient outcome (**Figure 4**).

From that point forward, efforts were made in most cooperative groups and QA centers to incorporate real-time object review into the QA process as often as possible and reasonable (Schwartz et al., 2009). For POG and then the COG, real-time RT object review became standard practice for clinical trials involving RT in Hodgkin's lymphoma (HL).

**FIGURE 5:**
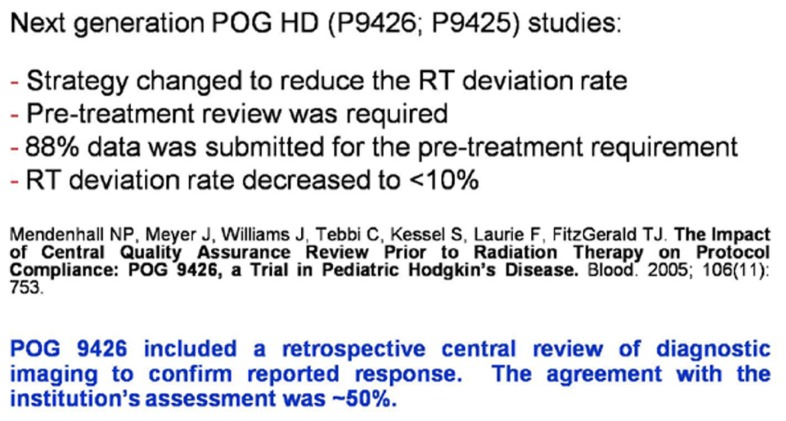
**Evolving review paradigm for pediatric HD trials**.

Continued progression of real-time review was evident in COG clinical trials 9425 and 9426 (HL high/low risk, respectively), where compliance for RT review pre-therapy was outstanding with a profound decrease in the RT study deviation rate (Tebbi et al., 2006; Schwartz et al., 2009; **Figure 5**).

**FIGURE 6:**
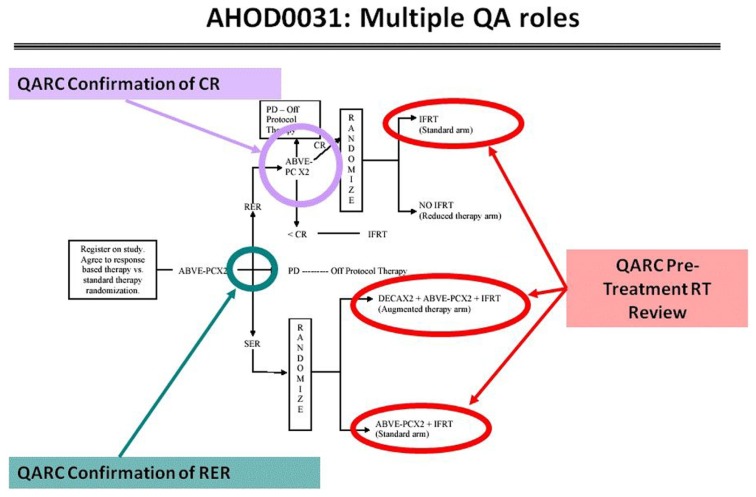
**Real-time review points in COG AHOD0031**.

These risk adaptive studies required the QARC to acquire the diagnostic images for response as part of the RT pre-treatment review. The concordance rate for response between site and central review was 50%, implying a clear need to integrate central review of image objects into the real-time review process. Accordingly, intermediate risk HL COG protocol AHOD0031 incorporated real-time review of diagnostic images for response as well as pre-treatment review of RT objects. Secondary and tertiary study randomization points were based on real-time central review of objects (Wolden et al., 2009; **Figure 6**). Currently open high-risk HL protocol AHOD0831 extends the process further as only selected sites of involvement that do not completely respond to chemotherapy undergo RT. Recent review of RT data from AHOD0031 confirms that pre-treatment review of RT treatment objects can significantly limit deviations on study (Dharmarajan et al., 2012a,b).

The evolution of real-time review strategies for diagnostic radiology and radiation oncology has been the culmination of process improvements in digital imaging and RT data transfer. The ACR through the RTOG and the ACRIN has made extraordinary contributions to the efforts of digital data transfer with digital transfer systems and web-based media for institutional credentialing and clinical trial conduct. ACRIN has successfully managed individual clinical trials accruing more than 50,000 patients per trial with advanced technology imaging for breast, lung cancer, and glioblastoma studies, as well as additional significant contributions to the other cooperative groups. In collaboration with members of the ITC, the RTOG has provided the infrastructure for digital radiation oncology data transfer as well as the institutional credentialing mechanisms with the RPC. These mechanisms have served to promote the process and availability of real-time object review to ensure the images and intended treatment plan are study compliant. The CALGB imaging core center likewise maintains a very similar process.

In spite of the clear success of these processes, including real-time reviews, and the improvements offered to clinical trial investigators, there is a perception that the overall QA process is expensive, excessive, and often unnecessary. Arguments are made that attempts to eliminate deviations confound clinical care as the process does not reflect the activities of daily clinical practice and deviations will be equally distributed among all arms, therefore not affecting study outcome. There are arguments to the contrary. The data suggest that patient outcome and uniform treatment delivery on study is likely best served by creating QA systems that ensure protocol compliant treatment strategy prior to treatment delivery. Through a multifaceted approach that incorporates education, credentialing and reviews, QA strategies can be tailored to the requirements of a particular protocol.

The recent and current COG Wilms'/renal studies provide examples of how pre-determined processes can provide clear direction for study success as well as opportunities for process improvement. As in most modern clinical trials, there are several endpoints imbedded into trial objectives. Patients are stratified into low-, intermediate-, and high-risk groups. High-risk patients with pulmonary nodules at presentation are treated with chemotherapy. Pulmonary images at presentation and week 6 of therapy are centrally reviewed through digital media in real time. If there is a complete response defined by central review, patients do not receive pulmonary RT. This process has worked exceptionally well and all follow-up images are collected for outcome analysis. Interestingly, there has been an unanticipated issue with anatomical distortion generated by the primary renal disease in the fused CT/planning studies requiring sagittal reconstruction. This has been responsible for many unanticipated RT deviations as traditional anatomic radiation treatment guidelines appear less consistent with targets definitions designed using three-dimensional objects. Three-dimensional planning tools are important in designing the inferior aspect of the pulmonary volume for whole lung therapy and the fused pre-operative CT images successfully predict for the inferior aspect of the renal target. Moving forward, the QA strategies would include pre-treatment review of objects and the expanded use of atlases, web-based interaction tools, and real-time review of objects. Clinical trial processes need to be adaptive in nature and, as in AHOD0031, be able to implement change during a study to insure study objectives. Reviews of processes during clinical trials will serve to improve study performance as adjustments are made to accommodate for problems identified during the conduct of the study.

Altruism with the intent of cost containment can lead to unexpected consequence and limitations in data availability. The CALGB completed an outstanding series of breast cancer clinical trials defining the use of dose dense chemotherapy including studies defining the potential role of taxol in node positive breast cancer patient care (Citron et al., 2003; Henderson et al., 2003).

In 1997, seminal publications revealed a survival advantage to node positive breast patients receiving RT (Overgaard et al., 1997; Ragaz et al., 1997). These and subsequent studies altered the landscape of therapy and changed the perception of local therapy in the treatment community. A retrospective review of patients treated on the CALGB trials revealed an extraordinary diversity of radiation dose and volume on patients receiving RT as there were no specific RT guidelines imbedded into the study. There was also a trend for patients who received taxol to have been treated with RT, thus complicating study interpretation. The retrospective review of data was difficult to accomplish as the primary trial objectives had been met and only limited data sets could be collected in retrospect as many patient files at participating institutions had been relocated and stored in various formats with limited retrieval capability. If data sets were collected as part of the protocol (not necessarily reviewed but stored), we would have been able to query the data and review the information in a timely manner (Sartor et al., 2005).

More recent events have further promoted the potential importance of breast cancer volumetric datasets. In the past several years multiple studies have addressed the question of the importance of axillary staging and therapy. National Surgical Adjuvant Breast and Bowel Project (NSABP) study B32 evaluated breast cancer patients treated with either axillary dissection or sentinel lymph node staging procedures and demonstrated that more limited axillary surgery with sentinel staging had equivalent patient outcome to axillary dissection. ACOSOG protocol Z0011 demonstrated that more extended axillary surgery may not be indicated even in node positive patients receiving RT. Study MA-20 conducted by NCI-Canada demonstrated the statistical importance of regional RT in node positive breast patients.

These studies all point to important issues in radiation oncology. However, no study collected volumetric treatment data. Modern radiation oncologists need to establish common ground as to how they will contour axillary structures and agree upon techniques for therapy to cover the volume, provide conformal avoidance to structures such as the axillary vessels and brachial plexus, and limit sequelae from radiation management. If volumetric RT treatment data were submitted on these studies coupled with outcome imaging from relapse, radiation oncologists could review these data and adjudicate strategies for RT protocol design moving forward. In three-dimensions many of the lymph node volumes at risk are in the anterior plane and often partially treated in breast fields. However, the posterior axilla abuts the latissimus muscle and data from the University of Florida does suggest regional control is improved in patients with additional posterior fields (Chang et al., 2007).

Our challenge in this time of finite resources will be to ensure data integrity for future analyses and to do this within the confines of defined resources. Gathering data for the future has a cost in terms of simple things similar to checking data integrity to make sure that the data will be useful at a later point in time.

## EDUCATION

The clinical trial QA programs assure quality and implement quality improvement (QI). These strategies include face to face and on-line workshops and training; on and off-site audits; web-based learning modules, resources, and tools; and fellowships. The QA centers provide education to a varied audience including the public, patients, researchers, and research staff. Providing on-going education for clinical researchers and staff strengthens the research and is an aspect of the International Conference on Harmonisation (ICH) good clinical practice quality standard.

## QUALITY ASSURANCE OF FUTURE CLINICAL TRIALS

Quality assurance for future clinical trials will seamlessly incorporate imaging, RT, pathology, and patient outcome data likely with a common informatics platform. The current QA centers will integrate under a unified global structure for imaging and RT. Four guiding principles for QA and informatics cooperative group support have been established. These principles are: (1) The QA process includes multiple activities starting with protocol development and including assessments of institutional capabilities and data quality; (2) Harmonization of QA processes and standards is needed to improve the efficiency and effectiveness of the QA process across the cooperative group network; (3) The IT systems that support imaging and RT QA should be interoperable and compliant with existing standards for protecting patient privacy and ensuring data integrity; and (4) Imaging and RT data collected in the QA process are important scientific resources. From the lessons learned, the QA processes and QI tools will be enhanced to promote efficiency and effectiveness. The funding source for this new QA system will originate from a single source independent of a specific cooperative group. Tissue banks will be funded separately, however, there is an expectation that digital pathology objects will be linked into review systems to expedite new biomarkers.

Imaging/Radiation Oncology Core Group will move toward building a fully integrated system that includes all of the current organizations in this QA system. Each of these organizations has their own software, work processes, and procedures in place. Innovation will come from within the group. Each of the IROC entities has functioning systems designed for the services and studies they support. To the outside world, these appear redundant; Insiders would likely argue that although they appear similar, they are uniquely designed and processes have been built around them so that each entity functions efficiently. Initially, these systems will be left in place and interoperability will be established with the goal of empathy with user requirements (usercentric). With a user friendly interface the investigator will be able to submit data and receive a receipt that the data reached its destination complete and not corrupted. Today's technologies yield a rich array of possibilities for collaboration and data exchange that can be maximized to support the heterogeneous community of clinical trial participating sites. Thus it is important to support all commonly accepted data acquisition and exchange strategies. It is relatively straightforward to take any of the common strategies and build functionality into it that validates the data and brings it into an organization's legacy system for further processing, review and archiving. In the case of QARC, there is the MAX database which functions as an operating system for the QARC staff and for the off-site reviewers who review clinical trial data from remote sites. As we move toward integration, each center will contribute their imbedded strength in informatics to a common platform.

The single portal with a friendly, functional interface benefits the user with one site for data submission. They can submit data using various “standard” transfer mechanisms at first and more sophisticated systems as the site develops. Investigators will access a single secure portal; identify their target organization and transmission method prior to the data submission. They will be informed immediately whether the data transfer was successful. This same interface will notify the target organization of data waiting to be further validated and utilized. The organization assigned to process, manage and/or review the data will retrieve it, verify that it is not corrupt and that it is the correct data for the particular protocol requirements. The data will then be available to import for processing and export to a shared repository. This will allow partner organizations to have access to the data immediately and will eliminate redundant processes.

Investigators will have access to the integrated database for evaluation of clinical trial objects. During the course of a clinical trial, data will be protected with limited access through traditional mechanisms established by the cooperative groups. Once trials are closed and the data statute of limitations is established, de-identified data can be made available to national and international investigators for secondary data analyses.

## EVALUATE AND PARTICIPATE IN NEW OPPORTUNITIES

Many clinical trial participants point toward redundancies in process and data submission as barriers to clinical trials involvement. Although credentialing centers such as the RPC, RTOG/ITC, ACRIN, and QARC have worked well together to integrate function and synergize credentialing mechanisms, investigator perceptions are often influenced by these and other credentialing processes. Due to the issues and regulations in daily clinical practice, incorporating clinical trial objectives is perceived as a challenge. The landscape is changing and there may be opportunity to re-engineer credentialing strategies in manners not previously anticipated. This will include an expanded use of registration trials for data collection and management.

In Massachusetts (MA), ACR credentialing is now required if a radiation oncology system is going to manage a new and/or satellite treatment facility and insurance companies are beginning to evaluate the use of credentialing mechanisms for reimbursement of advanced treatment technologies. Included in this strategy is a new system of IMRT approval which requires normal tissue volume metrics for validation of the use of this technology. This was recently approved by Blue Cross/Blue Shield of MA and several in-state companies are reviewing this program and will likely adopt this and other similar reimbursement strategies moving forward. This may provide institutional incentive for credentialing as the process of credentialing could serve more than one purpose. A synergistic strategy could be put into place to use the identical credentialing strategy to perform multiple functions. For example, approval of the RPC for beam output (optically stimulated luminescent dosimeter/thermoluminescent dosimeter, OSLD/TLD) measurement coupled review of department processes by the ACR/American. Society for Radiation Oncology (ACR-ASTRO) including remote case review would permit reciprocal credentialing for low tier participation in the clinical trials process. This can be expanded to sub-specialty based practice including pediatrics, gynecology, and others on an as-needed basis with periodic re-credentialing as appropriate. The tools currently in use and to be developed by the QA center would be an invaluable resource to this process in development. They would establish a nimble and potentially interactive infrastructure for credentialing and validating dose volume metrics for IMRT and advanced technology treatment delivery as well as the real-time review. This could serve to both credential institutions for clinical trials participation and validate the credentialing process for ACR and other regulatory agencies. This could mature into a tiered strategy with RT-specific questions requiring more rigorous review by the QA centers including dose/volume review with motion management. The opportunities for process improvements in cancer care are boundless working in concert with an integrated IT platform.

Building an integrated IT network including pathology will permit evaluation of other opportunities perhaps even beyond oncology. Many disciplines, including cardiology, critical care, neuroscience, etc. will benefit from integrated platforms including imaging and patient outcome data. Experts in late effects and many associated with the developing area of interventional oncology will be able to use integrated platforms joined with data from molecular oncology leading to discovery and the development of novel treatment strategies including the international community. This is a leadership moment for all involved and an opportunity to build a system that can lead and define the cancer care management of the future. Building upon the established strengths of the current QA systems, an integrated informatics and QA platform can be built to make the entire clinical trials program greater than the sum of the parts and serve the oncology clinical trials community by facilitating processes required for clinical trials execution.

## Conflict of Interest Statement

The authors declare that the research was conducted in the absence of any commercial or financial relationships that could be construed as a potential conflict of interest.
